# A *de novo* 2.78-Mb duplication on chromosome 21q22.11 implicates candidate genes in the partial trisomy 21 phenotype

**DOI:** 10.1038/npjgenmed.2016.3

**Published:** 2016-03-02

**Authors:** James D Weisfeld-Adams, Amanda K Tkachuk, Kenneth N Maclean, Naomi L Meeks, Stuart A Scott

**Affiliations:** 1 Division of Clinical Genetics and Metabolism, Department of Pediatrics, University of Colorado School of Medicine, Aurora, CO, USA; 2 Medical Genetics Clinic, Children’s Hospital Colorado, Aurora, CO, USA; 3 The Linda Crnic Institute for Down Syndrome, University of Colorado, Aurora, CO, USA; 4 Department of Genetics and Genomic Sciences, Icahn School of Medicine at Mount Sinai, New York, NY, USA

## Abstract

Down syndrome (DS) is the most common genetic cause of intellectual disability (ID) and in the majority of cases is the result of complete trisomy 21. The hypothesis that the characteristic DS clinical features are due to a single DS critical region (DSCR) at distal chromosome 21q has been refuted by recently reported segmental trisomy 21 cases characterised by microarray-based comparative genomic hybridisation (aCGH). These rare cases have implicated multiple regions on chromosome 21 in the aetiology of distinct features of DS; however, the map of chromosome 21 copy-number aberrations and their associated phenotypes remains incompletely defined. We report a child with ID who was deemed very high risk for DS on antenatal screening (1 in 13) and has partial, but distinct, dysmorphologic features of DS without congenital heart disease (CHD). Oligonucleotide aCGH testing of the proband detected a previously unreported *de novo* 2.78-Mb duplication on chromosome 21q22.11 that includes 16 genes; however, this aberration does not harbour any of the historical DSCR genes (*APP*, *DSCR1*, *DYRK1A* and *DSCAM*). This informative case implicates previously under-recognised candidate genes (*SOD1*, *SYNJ1* and *ITSN1*) in the pathogenesis of specific DS clinical features and supports a critical region for CHD located more distal on chromosome 21q. In addition, this unique case illustrates how the increasing resolution of microarray and high-throughput sequencing technologies can continue to reveal new biology and enhance understanding of widely studied genetic diseases that were originally described over 50 years ago.

## Introduction

Down syndrome (DS) is one of the prototypical disorders of human aneuploidy and copy-number variation based on its characteristic clinical presentation, the identification of trisomy 21 as the hallmark cytogenetic abnormality, and the continued study of chromosome 21 candidate genes for correlation with specific DS clinical features. The hypothesis that most aspects of the DS phenotype are owing to a single DS critical region (DSCR) at distal chromosome 21q was originally proposed in the 1970s.^1–3^ However, the continued advances in molecular cytogenetic technologies (e.g., fluorescence *in situ* hybridisation (FISH) and microarray-based comparative genomic hybridisation (aCGH)) have enabled greater resolution of segmental trisomy 21 aberrations and subsequent correlation studies between candidate genes and specific DS clinical features. Notably, a DSCR thought to be responsible for key DS clinical features has been defined by a region on chromosome 21q22.12-q22.2 containing *DSCR1* (*RCAN1*), *DYRK1A*, *DSCAM* and *APP*.^4–6^ However, recent studies of segmental trisomy 21 cases characterised by FISH and/or aCGH indicate that different features of the DS phenotype are likely attributable to several distinct genomic regions on chromosome 21 and not just a single DSCR.^7,8^

Despite the recent advances in ascribing DS clinical features to candidate genes and regions,^7,8^ the map of chromosome 21 copy-number aberrations and their associated DS clinical features remains incompletely defined. As such, the paucity of informative segmental trisomy 21 cases reported in the literature prompted our case report of a unique *de novo* 2.78-Mb duplication of chromosome 21q22.11 in a patient with a partial trisomy 21 phenotype. This case offers valuable insights into previously under-recognised candidate genes and regions potentially implicated in features of the DS phenotype.^9^

## Results

### Prenatal clinical history

The patient was born to a 36-year old mother, whose pregnancy had been calculated as high risk for DS (1 in 13) based on maternal age and an abnormal maternal quad screen (alfa-fetoprotein (AFP) 16.3 ng/ml, 0.47 MOM; hCG 75,636 mIU/ml, 0.74 MOM; uE3 0.61 ng/ml, 0.74 MOM; DIA 372 pg/ml; 1.72 MOM) at 15 weeks gestation; however, amniocentesis was declined. A two-vessel umbilical cord, abnormal fifth digits bilaterally, and normal cardiac anatomy were noted on antenatal ultrasound at 16 weeks gestation.

### Postnatal clinical evaluation

**Figure 1:**
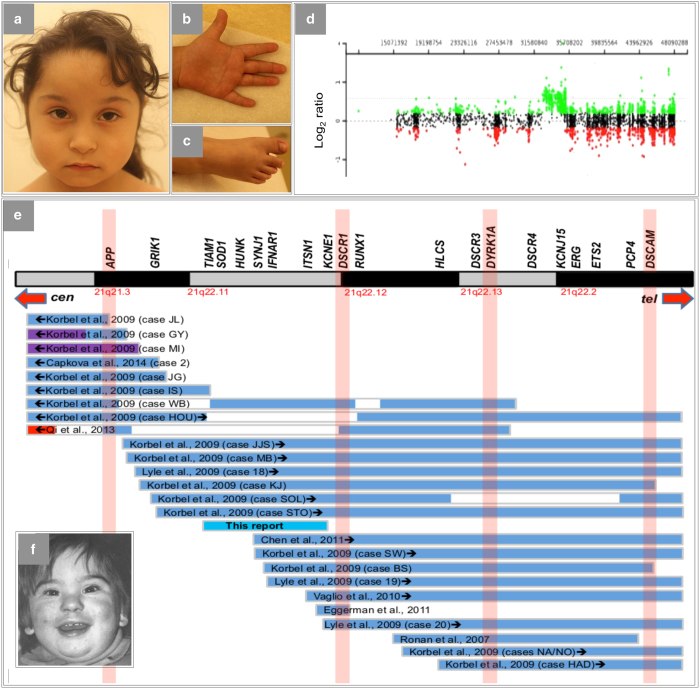
Phenotypic features of the proband and the patient reported by Huret *et al*.,^23^ and an illustration of the 21q21.2-q22.2 region highlighting the identified 2.78-Mb duplication and other reported segmental 21q duplication cases characterised by aCGH. (**a**–**c**) The proband at 5 years of age. Notable features include upslanting palpebral fissures, flat facies, prominent epicanthal folds and a flat nasal bridge. She also has bilateral single palmar creases and wide interspaces between the hallux and second digits on both feet. She has an ID, but lacks the ‘happy personality’ seen in many children with DS. Brachycephaly, protruding tongue and CHD were not present. (**d**) Oligonucleotide aCGH results of chromosome 21 in the proband. Coloured dots represent oligonucleotide aCGH probes plotted by their log_2_ ratios. The 2.78-Mb duplication at 21q22.11 is identifiable by the dense green probes with an average log_2_ ratio of ~0.6, indicating a single copy-number gain. (**e**) Chromosomal location of the 2.78-Mb duplication identified in the proband (light blue bar) compared with other reported interstitial copy-number aberrations characterised by aCGH with at least one breakpoint within 21q21.3-q22.2. Blue bands indicate duplications (partial trisomy), purple bands indicate triplications (partial tetrasomy) and red bands indicate deletions (partial monosomy). Black arrows indicate aberrations that extend proximally (left arrow) or distally (right arrow) beyond the limits of the region depicted in the figure. Genes previously identified as key critical genes in the overall DS phenotype are indicated by vertical pink translucent bars. (**f**) A French patient reported by Huret *et al.* in 1987 and photographed at 18 months of age has a similar facial appearance to our patient, lacked CHD and had a cytogenetically undetectable 21q duplication that likely involved *SOD1* (see Discussion). Figure 1f reproduced from ref. 23 with permission from Springer, copyright 1987.

The patient was referred to Medical Genetics at 4 years of age with physical features suggestive of DS. Height, weight and head circumference at most recent follow-up (age 5 years) were on the 3rd, 43rd and 30th centiles for age, respectively. Examination revealed a round, flat face with upslanting palpebral fissues, prominent epicanthae, flat nasal bridge (Figure 1a) and mild macroglossia. Ears had a normal appearance. Hands were broad and small with mild bilateral fifth digit clinodactyly and bilateral single transverse palmar creases, and feet showed wide interspaces between first and second toes bilaterally (Figure 1b,c). Despite the presence of mild to moderate developmental delays, the family declined a formal neuropsychologic characterisation of developmental deficits. Echocardiography showed no evidence of congenital heart disease (CHD). The patient lacked the happy, sociable affect observed in many children with DS. There was no history of intestinal atresia or other gastrointestinal malformations. Thyroid studies were normal, as was her tone, with no abnormal joint laxity or herniae.

### Clinical cytogenomic and molecular genetic analyses

Cytogenetic testing demonstrated a 46,XX normal female karyotype; however, aCGH detected a 2.78-Mb duplication of 21q22.11 (chr21:32,583,901–35,355,969; hg19) that included 16 genes (*TIAM1*, *SOD1*, *HUNK*, *MRAP*, *URB1*, *SYNJ1*, *C21ORF59*, *OLIG2*, *OLIG1*, *IFNAR2*, *IFNAR1*, *GART*, *SON*, *DONSON*, *CRYZL1* and *ITSN1*; Figure 1d). This region has only minimal overlap with smaller copy-number variations reported among healthy individuals in the Database of Genomic Variants,^10^ and no duplications of comparable size or coordinates are currently reported in the ClinGen^11^ and DECIPHER^12^ databases. The 21q22.11 aberration was confirmed in the proband by interphase FISH but not detected in either parent, indicating that the 2.78-Mb duplication occurred *de novo* in the proband. Metaphase FISH analysis was not possible to perform so it is not currently known whether this duplication is in tandem or inserted elsewhere in the genome. Taken together, these data indicated that the *de novo* 2.78-Mb duplication is pathogenic.

In addition, aCGH of the proband also identified a 31.0-kb deletion of 4p16.2 (chr4:5,564,364–5,595,303; hg19) that included exons 17–22 of *EVC2*. This deletion was confirmed and subsequently detected in the healthy father by qPCR, suggesting that it is a paternally inherited loss-of-function allele for autosomal recessive Ellis van Creveld syndrome (EVCS). A search for physical signs of EVCS, which includes acromelic dwarfism, polydactyly, CHD, oral frenulae and dental/nail anomalies, was negative. In addition, Sanger sequencing of the *EVC2* coding region in the proband did not detect any pathogenic mutations or variants of uncertain significance. Weyers acrofacial dysostosis (WAD) is characterised by dental/nail abnormalities and postaxial polydactyly, and is caused by dominant single-nucleotide mutations in the 3′ region of the last exon of *EVC2*,^13–15^ which escape nonsense-mediated messenger RNA decay and encode a dominant negative mutant EVC2 polypeptide.^13,14^ Furthermore, no multiexon deletions of *EVC2* have been previously reported among patients with WAD, further supporting the identified 31.0-kb deletion as a recessive allele for EVCS in the proband and father.

Although stature was short, the proband was taller than that would be expected for EVCS and approximated the 80th centile in height for females with DS. Parents and the younger sister of the proband showed no facial or other physical features suggestive of DS or EVCS/WAD, and there was no family history of learning disability.

## Discussion

We report the case of a child with clear neurodevelopmental and partial dysmorphologic features of DS, but without CHD, and who harbours a previously unreported *de novo* 2.78-Mb duplication of chromosome 21q22.11. Importantly, this aberration does not include *APP*, *DSCR1*, *DYRK1A* or *DSCAM*, which further argues against the hypothesis of a single DSCR responsible for the primary features of DS;^4–8^ however, the unique size and location of this duplication also supports a role for overexpression of other previously under-recognised genes in the aetiology of DS. To frame this aberration in context with other reported segmental trisomy 21 aberrations, previously reported patients with DS or partial DS phenotypes and interstitial duplications distal to 21q21.3 characterised by aCGH^6–8,16–19^ are summarised in Figure 1e. We excluded a recently reported case of a partial 21q duplication in an adult^20^ as the phenotype was likely confounded by a co-existent 2.2-Mb deletion of 7q36 in that patient.

One of the first informative cases of segmental trisomy 21 characterised by aCGH involved a maternally inherited 4.3-Mb duplication of 21q22.13-q22.2 that included *DRYK1A*, but not *DSCR1* or *DSCAM*.^6^ The mother and elder daughter were both of short stature, had mild learning disability and had a craniofacial phenotype consistent with DS; the younger daughter, who died in the neonatal period, also had characteristic facial features. Notably, neither daughter nor the mother had CHD, prompting the conclusion that the DS facies is dependent on duplication of *DYRK1A* and that a more telomeric region (including *DSCAM*) is likely implicated in the characteristic CHD observed in ‘typical’ DS.

Additional segmental trisomy 21 cases analysed by BAC aCGH further ruled out a single DSCR being responsible for the major features of DS and narrowed many DS phenotypes to a region between 34 and 41 Mb on chromosome 21.^7^ This important case series also indicated that short stature and abnormal dermatoglyphics were likely due to gene regions lying outside of this interval.^7^ Similarly, Korbel *et al*.^8^ assessed the clinical phenotypes of 30 patients with segmental trisomy 21 and correlated clinical features with duplications defined by high-resolution oligonucleotide aCGH, which narrowed a putative CHD region to a 1.77-Mb interval telomeric to *DSCR1* and *DYRK1A*, but including *DSCAM*, *RIPK4* and *ZBTB21*. Notably, critical regions for Alzheimer’s disease (AD) and intellectual ability (ID) were also interrogated, which supported a role for overexpression of *APP* in AD but not in ID. Interestingly, although both *DSCR1* and *DYRK1A* may have roles in the pathogenesis of ID, a necessary synergistic contribution of these genes to ID or CHD has not been definitively demonstrated, as some patients with ID and partial 21q duplication were trisomic for only one of either gene.^6,8^ The chromosomal map devised by Korbel *et al.*^8^ based on several patients with duplicated *APP* and normal copy number of other historically reported critical genes further suggests that more than one critical region for ID exists and argues against an essential role of *APP* in DS-associated ID. Moreover, a recently reported patient with an intestitial duplication involving *APP* and surrounding genes, but not extending distally as far as *DSCR1* and observed together with a small proximal 21q triplication, had mild speech delay but no other documented developmental delays, together with macroglossia and mild foot dysmorphology.^21^

Our patient does not have CHD, but has ID, a facial gestalt that clearly shares features with DS, as well as single palmar creases and wide digital interspaces. Importantly, her interstitial chromosome 21q22.11 duplication is 5.3 Mb distal to *APP*, and 55 kb, 3.4 and >6  Mb proximal to *DSCR1*, *DYRK1A* and *DSCAM*, respectively (light blue bar, Figure 1e). This relatively small duplication supports previous studies that refuted exclusive roles for increased dosage of *DSCR1*, *DYRK1A* and *DSCAM* in the pathogenesis of neurodevelopmental and dysmorphologic aspects of the DS phenotype^6–8^ and reveals additional candidate genes for DS clinical features. The absence of CHD in our patient corroborates previous studies that concluded that a critical region for CHD is located distal to the identified 2.78-Mb duplication of chromosome 21q22.11, possibly including *DSCAM* as previously suggested.^6,8^

Among the OMIM-annotated genes in the identified duplication, there are few clear candidates for genes that explain the elements of our patient’s phenotype. However, previous work has suggested that overexpression of *SOD1* may contribute to some aspects of DS, and it was demonstrated over 30 years ago that SOD1 activity in fetal brain is enhanced in trisomy 21.^22^ Soon afterwards, Huret *et al*.^23^ reported a male patient with many aspects of DS, including the characteristic facial gestalt and ID. The patient’s facial appearance (Figure 1f, reproduced with permission), was similar to that of our patient and neither had CHD. Notably, he was disomic for chromosome 21, but using superoxide dismutase enzyme assays, DNA studies and *in situ* hybridisation techniques, the authors concluded that he harboured a microduplication at 21q21-21q22.1 that included *SOD1* but was below the level of traditional karyotype resolution.^23^ In *Drosophila*, increased *SOD1* expression has been implicated in hyperphosphorylation of tau, which may serve as an important factor for AD susceptibility in children with DS.^24^ and abnormal *SOD1* expression may also perturb oxidative stress responses, which are protective against AD.^25^

Another candidate gene within the identified 2.78-Mb duplication that may also contribute to the DS phenotype when overexpressed is *SYNJ1*, mutations in which causes an autosomal recessive Parkinson’s disease phenotype.^26^ This is supported by a transgenic murine model study, which suggested that appropriate *Synj1* dosage is important for normal brain development and that overexpression of *Synj1* is implicated in the brain dysfunction observed in DS.^27^ Pucharcos *et al*.^28^ previously demonstrated in a mouse model that *itsn1* is expressed in both proliferating and differentiating neurons, and proposed that *ITSN1* overexpression may contribute to DS pathogenesis.

In conclusion, the identified 2.78-Mb duplication at 21q22.11 supports roles for *SOD1*, *SYNJ1* and/or *ITSN1* overexpression in the partial trisomy 21 phenotype, specifically correlating with characteristic DS facial features, hand and foot dysmorphology, and ID. This unique clinical case again underscores the utility of cytogenomic approaches to the complex DS phenotype as well as other Mendelian disorders, and indicates that the ongoing advances in genomic technologies, including whole-genome sequencing, will continue to clarify unanswered questions in clinical genetics as well as illuminate new hypotheses and uncertainties in the evolving discipline of genomic medicine. Each newly identified partial 21q duplication patient should be reported in order to advance our understanding of genotype–phenotype correlations in the DS phenotype.

## Materials and methods

### Clinical evaluation

Clinical evaluation was performed at the Medical Genetics clinic at Children’s Hospital Colorado by a board-certified clinical geneticist and paediatrician (J.D.W-A.) and genetic counselor (A.K.T.). Informed consent was obtained from the mother of the patient for their participation in this study, including the use of medical information and the publication of the patient's photograph (Figures 1a–c). Methods were performed in accordance with relevant regulations and guidelines.

### Clinical cytogenetic and molecular genetic testing

Clinical cytogenetic and cytogenomic testing included karyotype analysis performed at the Colorado Genetics Laboratory (CGL) at the University of Colorado, and aCGH testing using the 400 K CMA-COMP array v9.1 (Agilent Technologies, Inc., Santa Clara, CA, USA) performed at the Baylor Miraca Genetics Laboratories. The identified chromosome 21q22.11 duplication was further interrogated at the Baylor Miraca Genetics Laboratories by interphase FISH using the RP11-484I12 BAC probe. The identified chromosome 4p16.2 deletion was interrogated at the DNA Diagnostic Laboratory at the University of Colorado Denver using a dual Taqman probe quantitative PCR (qPCR) assay (Roche Diagnostics Corporation, Indianapolis, IN, USA) with 4p16.2 region-specific primers (forward: 5′- GTGCACTCACATTGCACCAT-3′; reverse: 5′- CATGACTCTGTCTTGCCTGGT-3′) and a Universal Probe (5′- CATCCAGC-3′). In addition, *EVC2* Sanger sequencing was performed at the DNA Diagnostic Laboratory at the University of Colorado, Denver, using standard protocols.
