# A *de novo* 2.78-Mb duplication on chromosome 21q22.11 implicates candidate genes in the partial trisomy 21 phenotype

**DOI:** 10.1038/npjgenmed.2017.1

**Published:** 2017-08-25

**Authors:** James D Weisfeld-Adams, Amanda K Tkachuk, Kenneth N Maclean, Naomi L Meeks, Stuart A Scott

**Correction to:** *npj Genomic Medicine* (2016) **1**, 16003; doi:10.1038/npjgenmed.2016.3; published online 2 March 2016

The following text was missing from the paper and has now been added:

In the Materials and methods section:

‘Informed consent was obtained from the mother of the patient for their participation in this study, including the use of medical information and the publication of the patient’s photograph (Figures 1a–c). Methods were performed in accordance with relevant regulations and guidelines.’

In the caption of Figure 1:

‘Figure 1f reproduced from ref. 23 with permission from Springer, copyright 1987.’

These errors have now been corrected in the HTML and PDF versions of this Article.

